# Outcomes of Femoral Neck Fractures in Young Patients and the Factors Associated With Complications: A Multicenter Study From Malaysia

**DOI:** 10.7759/cureus.18110

**Published:** 2021-09-19

**Authors:** Jeffrey Jaya Raj, Ren Yi Kow, Kunalan Ganthel@Annamalai, Dinesh Earnest Kunasingh, Govind Kishen Panicker, Bee Chiu Lim, Chooi Leng Low

**Affiliations:** 1 Department of Orthopaedics, Hospital Kuala Lumpur, Kuala Lumpur, MYS; 2 Orthopaedics, Hospital Tengku Ampuan Afzan, Kuantan, MYS; 3 Orthopaedics, International Islamic University Malaysia, Kuantan, MYS; 4 Department of Orthopaedics, Hospital Kuala Lipis, Kuala Lipis, MYS; 5 Department of Orthopaedics, Hospital Sultan Haji Ahmad Shah, Temerloh, MYS; 6 Clinical Research, Hospital Tengku Ampuan Afzan, Kuantan, MYS; 7 Radiology, Hospital Tengku Ampuan Afzan, Kuantan, MYS

**Keywords:** neck of femur fracture, young adult hip, surgical fixation, bipolar hemiarthroplasty, total hip replacement (thr), screw fixation

## Abstract

Background and objective

Femoral neck fractures are relatively rare in young adults, but they result in prolonged loss of function in these patients, thereby placing a huge burden on a country’s healthcare and economy. Femoral neck fractures in young adults are normally treated with head salvage surgery. However, primary head replacement surgeries have been gaining traction recently to expedite the recovery of these patients. In this study, we aimed to investigate the outcomes in young patients with femoral neck fractures and factors associated with their complications.

Patients and methods

Patients with femoral neck fractures who underwent surgery in three tertiary hospitals [Hospital Tengku Ampuan Afzan (HTAA), Hospital Sultan Haji Ahmad Shah (HOSHAS), and Hospital Kuala Lipis] in Pahang state in Malaysia were reviewed and included in this study. The demographic profile of the patients, injury patterns, intervention details, functional outcomes, and complications were analyzed. The comparison between the sociodemographics, clinical assessment, and complication outcomes was analyzed using statistical software.

Results

The complications were associated with the mechanism of injury, capsulotomy, and type of fixation. A total of 46 patients were included in the study. Most of the patients were found to have severe displacement based on Garden and Pauwels classification. The majority of the patients underwent femoral head salvage surgeries. Almost half of the patients sustained complications and were unable to achieve weight-bearing status at six months postoperatively.

Conclusion

Despite a relatively short follow-up period in our study, femoral neck fractures in young adults were found to be associated with a high rate of complications. Primary head replacement surgeries should be considered in high-risk patients with femoral neck fractures.

## Introduction

Femoral neck fractures have a high incidence rate among the general population, accounting for 3.6% of all fractures [1]. They have a considerable impact on a country's healthcare and economy, due to the associated high morbidity and mortality rates [2-8]. These fractures are fairly common among the elderly population, which have a diminished bone density, making them prone to femoral neck fractures [2-8]. In contrast, femoral neck fractures are not common in young patients, with only up to 10% of such fractures sustained by younger adults [5]. Femoral neck fractures among younger patients usually occur due to high-velocity mechanisms of injury such as road traffic accidents or falls from height [6-10].

Unlike the elderly patients, young patients (<60 years) normally have good bone quality and longer life expectancy, and hence the focus of treatment for femoral neck fractures in them is the preservation of natural hip anatomy and mechanics [5-14]. The recommended treatment for femoral neck fractures in young patients is femoral head-preserving surgery via internal fixation due to high demand and function [5-11]. Thus far, there is no consensus on the optimal implant options for these patients, which generally include fixation via multiple cancellous screw fixation, sliding hip screw, or proximal femoral nailing [5-13]. Nevertheless, femoral head-preserving surgeries come with a cost, with 5-20% of the patients reported to have complications such as non-union, avascular necrosis (AVN), malunion, surgical site infection, and implant failure, necessitating revision surgery or conversion to hip replacement surgery [15-24].

Hitherto, there is no reported data from Malaysia on young adults with femoral neck fractures. In light of this, we conducted a multicenter review of the clinical outcomes of femoral neck fractures among young patients in Pahang state. Pahang state, the largest state in Peninsular Malaysia, with a population of 1.67 million, is served mainly by three tertiary hospitals: Hospital Tengku Ampuan Afzan (HTAA), Kuantan; Hospital Sultan Haji Ahmad Shah (HOSHAS), Temerloh; and Hospital Kuala Lipis. Our study focused on the young patients' demographics and injury profiles related to femoral neck fractures, and their treatment as well as subsequent outcomes.

## Materials and methods

This was a cohort study involving all patients aged 18-60 years who underwent surgical interventions for femoral neck fractures at three tertiary centers in Pahang, Malaysia, between 1st January 2018 to 30th June 2020. The hospitals included in this study are HTAA in the Kuantan district, HOSHAS in Temerloh, and Hospital Kuala Lipis in the Kuala Lipis district. This study is registered under National Medical Research Registry (NMRR) and approved by the Medical Research and Ethics Committee (MREC), Ministry of Health (MOH), Malaysia.

All patients were reviewed, and those who fulfilled the inclusion criteria were included. All acute and referred cases were included in this study. Postoperatively, all patients were followed up at the clinic at two weeks, six weeks, three months, and six months. Those patients who presented with complications were treated accordingly upon presentation. The inclusion criteria were as follows: patients aged 18-60 years with femoral neck fractures, who underwent surgical intervention in any of the three centers mentioned above, with a minimum of six months of follow-up. Patients who were treated conservatively, those with iatrogenic femoral neck fractures, ipsilateral femoral shaft fractures, and those who did not fit the age criteria were excluded from this study. Patients with incomplete follow-up and missing data were also excluded from this study.

The relevant data were extracted from the clinical records, including the demographic profile of the patients (age, comorbidity), injury patterns (side of injury, fracture type based on Garden and Pauwels classification, time to surgery), intervention details (capsulotomy status, type of surgery, implant used), functional outcomes (weight-bearing status at six months postoperatively), and complications (AVN, non-union, implant failure, dislocation, and infection). The comorbidities taken into account were diabetes mellitus, hypertension, ischaemic heart disease, hyperlipidemia, and gout. The comorbidities were classified as present or absent for the purpose of statistical analysis. Similarly, the types of surgical intervention were also classified into head-preserving surgery and head replacement surgery.

All head-preserving surgeries were performed at these tertiary centers by senior registrars or orthopaedic surgeons with a minimum orthopaedic experience of three years. All screw fixations were performed percutaneously using the standard lateral approach on the traction table with three 6.5-mm cannulated screws placed in an inverted triangle pattern. Similarly, proximal femoral nailing and sliding of hip screws were performed in the standard manner with the open incision made to accommodate the blade and plate insertion. The head replacement surgeries were performed by orthopaedic surgeons or consultants with a minimum orthopaedic experience of six years. Both hemiarthroplasty and total hip replacement (THR) were done using the standard lateral (modified Hardinge) approach in the lateral decubitus position. All patients underwent hemiarthroplasty with Thompson hemiarthroplasty stem with bone cement implantation. Meanwhile, for THR patients, hybrid THRs were utilized with cemented femoral stem inserted and cementless acetabular cup anchored with screws.

Data were entered into and analyzed using SPSS Statistics version 25.0 (IBM, Armonk, NY). The results were presented in numbers and percentages for categorical variables. Mean and standard deviations were used to present continuous variables unless otherwise stated. As age was not normally distributed, the age groups were classified into "less than 50 years" and "more than or equal to 50 years" for statistical analysis; 50 years was used as the cut-off point as those patients were deemed young with better bone density [2,25]. Concurrently, time to surgery was classified into "less than or equal to five days" and "more than five days" for statistical analysis [15]. The comparison between the sociodemographics, clinical assessment, and complication outcomes was analyzed using Pearson's chi-square test or Fisher's exact test. A p-value of <0.05 was considered statistically significant.

## Results

A total of 46 patients fulfilled the inclusion criteria and were included in this study. Majority of the patients were from HOSHAS (n=18, 39.2%), followed by HTAA (n=14, 30.4%), and Hospital Kuala Lipis (n=14, 30.4%). The demographic data and injury patterns of the patients included are summarized in Table 1. The age of the patients ranged from 19 to 60 years, with a median age of 51.5 years (interquartile range: 17). The patients predominantly sustained femoral neck fractures after a fall (n=27, 58.7%), while some patients had fractures due to road traffic accidents (n=19, 41.3%). Most of the patients sustained left-sided femoral neck fractures (n=28, 60.9%). More than 80% of the patients (n=38, 82.6%) had underlying medical conditions.

In terms of injury pattern, there was no Garden I and Pauwels I femoral neck fractures in this study. A large number of the patients sustained displaced femoral neck fractures, with 80% of the patients having Garden III (n=23, 50.0%) and IV (n=14, 30.4%) injuries, and more than two-thirds of the patients sustained Pauwels III injuries (n=31, 67.4%). The most common form of surgical intervention was head salvage surgeries (n=32, 69.6%), compared to head replacement surgeries (n=14, 30.4%). More than half of the patients underwent screw fixation (n=25, 54.3%), followed by hemiarthroplasty (n=10, 21.7%), sliding hip screw (n=5, 10.9%), THR (n=4, 8.7%) and proximal femoral nail (n=2, 4.3%). Among these patients, there was an equal number of those who had undergone capsulotomy intraoperatively and those who had not undergone capsulotomy.

**Table 1:** Demographic data of the patients HTAA: Hospital Tengku Ampuan Afzan; HOSHAS: Hospital Sultan Haji Ahmad Shah; RTA: road traffic accident

Variables		Median (interquartile range)	N (%)
Age (years)		51.5 (17)	
Time to surgery (days)		4.5 (5)	
Center	HTAA		14 (30.4)
	HOSHAS		18 (39.2)
	Hospital Kuala Lipis		14 (30.4)
Mechanism	RTA		19 (41.3)
	Fall		27 (58.7)
Side	Left		28 (60.9)
	Right		18 (39.1)
Comorbidity	None		8 (17.4)
	Present		38 (82.6)
Garden	II		9 (19.6)
	III		23 (50.0)
	IV		14 (30.4)
Pauwels	II		15 (32.6)
	III		31 (67.4)
Fixation methods	Screw fixation		25 (54.3)
	Proximal femoral nail		2 (4.3)
	Sliding hip screw		5 (10.9)
	Hemiarthroplasty		10 (21.7)
	Total hip replacement		4 (8.7)
Capsulotomy	No		23 (50.0)
	Yes		23 (50.0)

At six months postoperatively, there were only 25 patients (54.3%) who regained their mobility (Table 2). A total of 22 patients (47.8%) developed complications after surgical intervention of the femoral neck fractures (Table 2). Most of the surgical interventions were complicated with AVN of the femoral neck (n=15, 32.6%), followed by non-union (n=5, 10.9%), and infection (n=2, 4.3%). The breakdown of the type of complications based on the injury pattern (Garden and Pauwels classification) is summarized in Table 3.

**Table 2:** Outcomes in patients at six months after surgery

Variables		N (%)
Weight-bearing status (at 6 months)	No	21 (45.7)
	Yes	25 (54.3)
Complication	None	24 (52.2)
	Avascular necrosis	15 (32.6)
	Non-union	5 (10.9)
	Infection	2 (4.3)

**Table 3:** Distribution of complications and fracture classification

Variables	No complication, n (%)	Avascular necrosis, n (%)	Non-union, n (%)	Infection, n (%)
Garden				
II	3 (30.0)	5 (50.0)	1 (10.0)	1 (10.0)
III	11 (50.0)	6 (27.3)	4 (18.2)	1 (4.5)
IV	10 (71.4)	4 (28.6)	0 (0.0)	0 (0.0)
Pauwels				
II	8 (50.0)	6 (37.5)	1 (6.3)	1 (6.3)
III	16 (53.3)	9 (30.0)	4 (13.3)	1 (3.3)

Upon univariate analysis, a significant association was found between complications and mechanism of injury (p=0.019), capsulotomy (p<0.001), and type of fixation (p<0.001) (Table 4). However, there was no significant association between complications and age (p=0.08), time to surgery (p=0.277), comorbidity status (p>0.95), and severity of injury pattern (Garden and Pauwels classification).

**Table 4:** Association between demographics, clinical assessment, and complications after fixation as per univariate analysis *Fisher’s exact test NA: not applicable; RTA: road traffic accident

Variables	Complication, n (%)		Pearson's chi-square/Fisher's exact test	P-value
	No	Yes		
Age (years)				
<50 years	8 (38.1)	13 (61.9)	3.069	0.080
≥50 years	16 (64.0)	9 (36.0)		
Mechanism of injury						
Fall	18 (66.7)	9 (33.3)	5.502	0.019
RTA	6 (31.6)	13 (68.4)		
Time to surgery (days)				
≤5 days	15 (46.9)	17 (53.1)	1.183	0.277
>5 days	9 (64.3)	5 (35.7)		
Capsulotomy				
No	5 (21.7)	18 (78.3)	17.076	<0.001
Yes	19 (82.6)	4 (17.4)		
Comorbidity				
None	4 (50.0)	4 (50.0)	NA*	>0.950
Present	20 (52.6)	18 (47.4)		
Fixation type				
Head salvage	11 (34.4)	21 (65.6)	13.349	<0.001
Head replacement	13 (92.9)	1 (7.1)		
Garden				
II	3 (33.3)	6 (66.7)	NA*	NA
III	11 (47.8)	12 (52.2)		
IV	10 (71.4)	4 (28.6)		
Pauwels				
II	8 (53.3)	7 (46.7)	0.012	0.913
III	16 (51.6)	15 (48.4)		

## Discussion

The incidence of femoral neck fractures in young patients is uncommon but often associated with surgical challenges and a high rate of complications [4-6]. Inconsistent with the literature, femoral neck fractures in our study cohort were attributed to high-energy trauma such as road traffic accidents and falls from height [5]. AVN and non-union of femoral neck fractures have been reported at a rate of 11-86% and 16-59%, respectively [8-12,16,17]. One study has demonstrated that acceptable results can be obtained with early, accurate fracture reduction and rigid internal fixation [11]. The authors reported that 76% of the patients recovered with good or satisfactory functional outcomes in a mean period of 25 months [11].

Schweitzer et al. have demonstrated that patients aged more than 53.5 years had a higher risk of developing AVN [14]. In contrast, we found no association between age and the rate of complications, owing to the fact that there was a higher percentage of patients aged more than 50 years who had undergone head replacement surgeries. In our series, the median time to surgery was 4.5 days, as the implants were not readily available and needed to be arranged. Consistent with the study by Gumustas et al., there was no significant association between the timing of surgery and complications in patients with femoral neck fractures [15]. More than the timing of surgery, achieving absolute anatomical reduction is of utmost importance in optimizing outcomes and function [5,6,8]. Koaban et al. have reported a 17.4% incidence of AVN over 10 years in young adult patients with femoral neck fractures, and it was associated with the mode of injury [6]. On the contrary, we found no such association in our study.

The reasons and risk factors associated with developing higher rates of complications post neck of femur fixation in the young patients may be attributed to vascular damage from the initial femoral neck fracture, poor quality of reduction or fixation of the fracture (restoring flow to the distorted arteries) and the elevated intracapsular pressure [8-12,16,17]. Unstable fixation leading to varus collapse and non-union due to failure of absolute anatomical reduction of femoral neck fractures may also contribute to the high complication rate seen post-fixation [17,18]. There should be a low threshold for open reduction and internal fixation via an anterolateral Watson-Jones approach or Anterior Smith-Petersen approach when percutaneous screw fixation methods fail to achieve an adequate absolute anatomical reduction of the neck of femur fractures [17,18].

The aim of preserving the native hip joint in a femoral neck fracture in the young patient comes at the cost of AVN (14%), severe femoral neck shortening (13-32%), and non-union (9%) [8-12,16,17]. All previous studies in the literature advocate the preservation of the native hip joint in young femoral neck fractures and recommend arthroplasty as a salvage procedure after the failure of head-preserving surgery, howbeit our study showed a high complication rate in young femoral neck fracture fixation, making head replacement surgery such as THR a viable option as primary treatment. This idea has been strengthened by a population-based study by Stockton et al., where they illustrated one-third of the patients required reoperation and 14% of the patients had to undergo conversion to THR after initial femoral head salvage procedures [23]. They advocate head replacement surgery as the first line of surgery to expedite the recovery of patients with femoral neck fractures [23].

Besides averting the risk of complications and subsequent reoperation, hip replacement surgeries also allow patients to fully weight-bear postoperatively and thus accelerate the recovery process and subsequent return to early function [17,23]. In contrast, head-preserving surgeries require a prolonged period of immobilization and non-weight-bearing until complete consolidation of the femoral neck fractures is achieved. In this study, we found that the risk of developing complications was significantly higher in patients who had undergone femoral head-preserving surgeries as compared to those who had undergone femoral head replacement. By reducing postoperative complications, the patients who had femoral head replacement surgeries were able to return to ambulation and resume normal activities of daily living.

The main disadvantage of a primary femoral head replacement surgery is the finite years of implant survival and risk of dislocation, making primary femoral head replacement surgery a subject of controversy as a treatment method in young patients with femoral neck fracture [25-27]. In spite of this, the advancement in implant design, superior highly cross-link polyethylene liners, and improved surgical techniques have contributed to longer survival rates in THR [25]. A meta-analysis by Mei et al. involving 3,219 THRs demonstrated a survival rate of up to 99.5% at 10-14 years, 84% at 15-19 years, 77% at 20-24 years, and 60% at 25-30 years [25]. With the average life expectancy of the Malaysian population hovering at 68.9 years, femoral head replacement surgery is a feasible option for patients aged 45 years and above [28].

Limitations of the study

Our study had several limitations. Primarily, the nature of retrospective data collection limits the standardization of the data collection and may have led to the potential omission of some important data. Nevertheless, retrospective data collection ensured that there was no bias in deciding the surgery of choice for the patients with femoral neck fractures. Despite collecting data from multiple centers over a period of two years, the small sample size limited the interpretation of the data, making multivariate analysis not possible. Additionally, a cut-off point of six months was inadequate to determine the survival rate of an implant in patients with femoral neck fractures. Despite these limitations, this was a first-of-its-kind study to be conducted in a southeast Asian country. Further multicenter, long-term, prospective studies should be conducted to verify our findings.

## Conclusions

Based on our findings, femoral neck fractures in young adults had a high rate of complications, especially AVN and non-union. The complications were associated with the mechanism of injury, capsulotomy, and type of fixation. We recommend head replacement surgeries, specifically THR, to be considered and offered as an important modality of management in young femoral neck fracture patients to avoid the high rate of complications and expedite early return to function for patients.
